# Unmyelinated nerve fibers in the human dental pulp express markers for myelinated fibers and show sodium channel accumulations

**DOI:** 10.1186/1471-2202-13-29

**Published:** 2012-03-19

**Authors:** Michael A Henry, Songjiang Luo, S Rock Levinson

**Affiliations:** 1 Department of Endodontics, University of Texas Health Science Center at San Antonio, San Antonio, TX 78229, USA; 2 Department of Comprehensive Dentistry, University of Texas Health Science Center at San Antonio, San Antonio, TX 78229, USA; 3 Department of Physiology and Biophysics, University of Colorado School of Medicine, Aurora, CO 80045, USA

## Abstract

**Background:**

The dental pulp is a common source of pain and is used to study peripheral inflammatory pain mechanisms. Results show most fibers are unmyelinated, yet recent findings in experimental animals suggest many pulpal afferents originate from fibers that are myelinated at more proximal locations. Here we use the human dental pulp and confocal microscopy to examine the staining relationships of neurofilament heavy (NFH), a protein commonly expressed in myelinated afferents, with other markers to test the possibility that unmyelinated pulpal afferents originate from myelinated axons. Other staining relationships studied included myelin basic protein (MBP), protein gene product (PGP) 9.5 to identify all nerve fibers, tyrosine hydroxylase (TH) to identify sympathetic fibers, contactin-associated protein (caspr) to identify nodal sites, S-100 to identify Schwann cells and sodium channels (NaChs).

**Results:**

Results show NFH expression in most PGP9.5 fibers except those with TH and include the broad expression of NFH in axons lacking MBP. Fibers with NFH and MBP show NaCh clusters at nodal sites as expected, but surprisingly, NaCh accumulations are also seen in unmyelinated fibers with NFH, and in fibers with NFH that lack Schwann cell associations.

**Conclusions:**

The expression of NFH in most axons suggests a myelinated origin for many pulpal afferents, while the presence of NaCh clusters in unmyelinated fibers suggests an inherent capacity for the unmyelinated segments of myelinated fibers to form NaCh accumulations. These findings have broad implications on the use of dental pulp to study pain mechanisms and suggest possible novel mechanisms responsible for NaCh cluster formation and neuronal excitability.

## Background

The human dental pulp represents an attractive model system for the study of pain and is a common site of disease and pain [1-3]. Toothache pain can be quite severe and even though pain perception involves an integrated construct based upon central and peripheral mechanisms, the peripheral components present within the dental pulp appear to be critically important to the acute pain experience since pulp removal typically provides a rapid and complete relief of pain [4,5]. Of special note when considering the usefulness of the dental pulp as a model system to study pain is the finding that the application of nearly all physiologic stimuli applied to the human pulp results in the sensation of pain [6-8].

The nerve fiber density within the human dental pulp is quite impressive [9] and multiple studies have characterized these fibers relative to the presence or absence of myelin with the use of the electron microscope. The results of these studies generally show that 70-90% of the fibers are unmyelinated [10,11]. This preponderance of unmyelinated fibers contrasts sharply with the results of other studies performed in experimental animals that suggest a more extensive innervation of the dental pulp by myelinated afferents (see Discussion). Taken together, these results suggest that many of the unmyelinated axons within the dental pulp originate from parent axons that are myelinated at more proximal locations.

Although the results of these animal studies provide considerable evidence for a thinning of pulpal afferents as they course from the trigeminal ganglion to the dental pulp, this possibility has not been specifically examined in humans. The present study examines the expression of neurofilament heavy (NFH) protein, a protein commonly expressed within sensory neurons that give rise to myelinated afferents [12], to test the hypothesis that many of the unmyelinated pulpal afferents within the human dental pulp originate from myelinated axons. Knowledge concerning the relative contribution of pulpal innervation from sensory neurons that give rise to either myelinated or unmyelinated peripheral nerve fibers is important since the fiber type strongly influences the characteristic quality of pain experienced following peripheral nociceptor activation [13-15].

Moreover, the examination of normal and diseased human dental pulp specimens has proven as a useful model system to examine changes in sodium channel (NaCh) expression seen in specimens associated with pain [16-18]. Results from these studies have included the identification of NaCh clusters at non-nodal sites in both normal and diseased/painful samples [16]. In this study we take the opportunity to characterize the fibers with NaCh accumulations at non-nodal sites to test the hypothesis that the unmyelinated segments of myelinated axons show an inherent ability to cluster NaChs. The identification of NaCh clusters in unmyelinated fibers would be important since this finding would imply novel mechanisms responsible for this cluster formation and with potential contributions to axonal excitability. Therefore, the purpose of this investigation was a characterization of fibers with NFH expression within the human dental pulp and of the NaCh clusters seen at non-nodal sites in these same fibers in an attempt to more fully understand the character of pulpal afferents and how these findings could potentially impact our understanding of pulpal pain mechanisms and pain mechanisms in general.

## Results

### Most fibers with PGP9.5 also express NFH/N52

**Figure 1:**
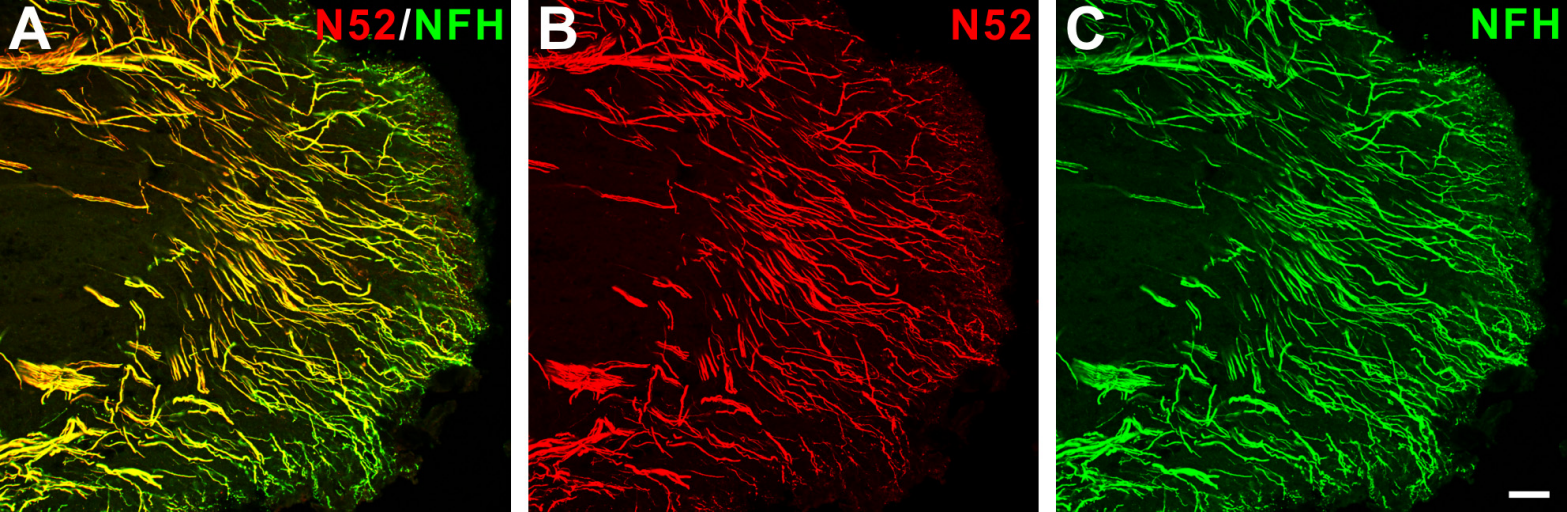
**Neurofilament 200 kDa expression is prominent in the human dental pulp**. A-C. Confocal micrographs showing nerve fibers identified by two different neurofilament 200 kDa antibodies [B, N52-mouse monoclonal; C, Neurofilament heavy (NFH)-chicken monoclonal] in the pulp horn of a normal human dental pulp. The overlapping of the N52 and NFH immunoreactivity appears yellow in the merged image (A). Scale bar, 50 μm.

**Figure 2:**
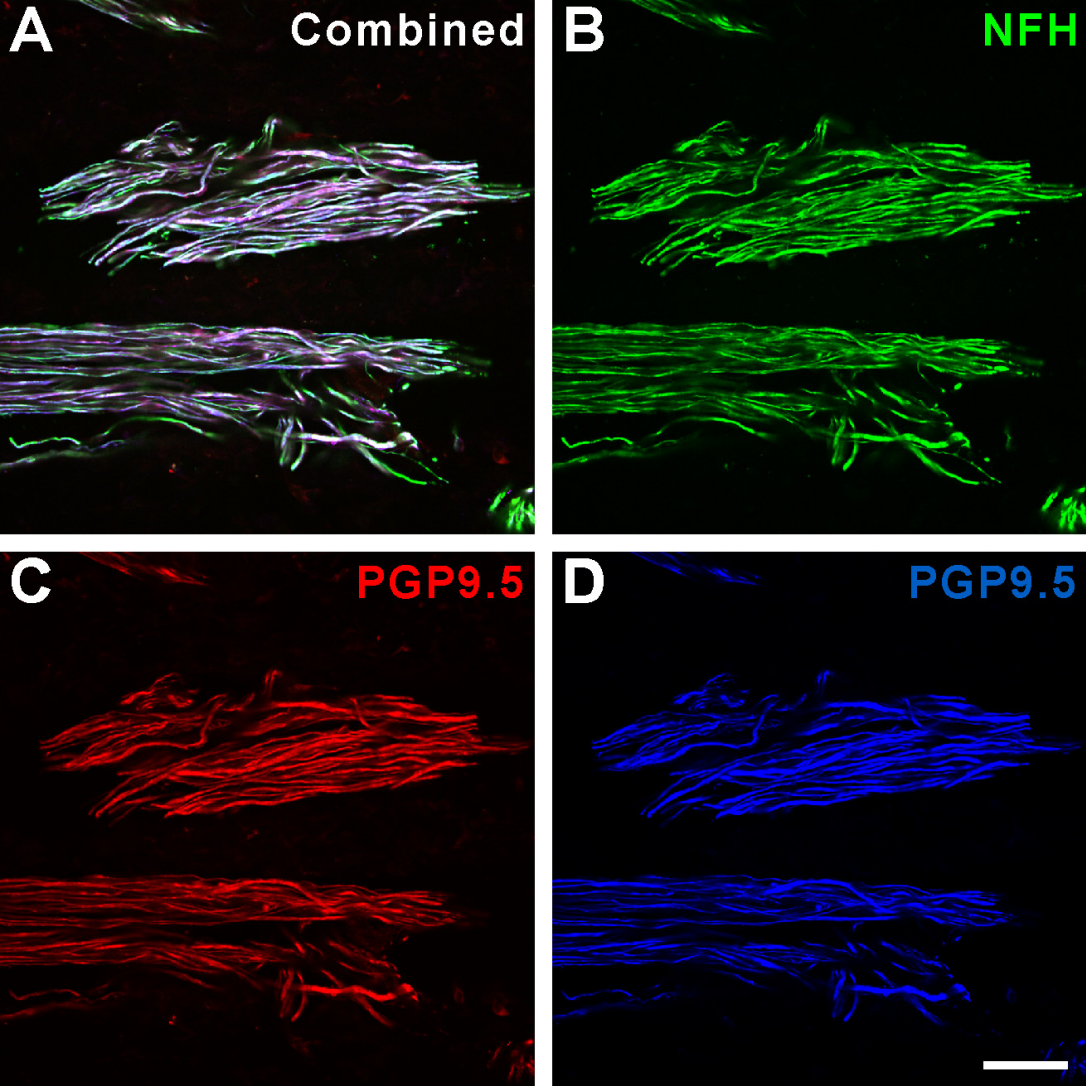
**Most nerve fibers in human dental pulps express both PGP9.5 and neurofilament heavy (NFH)**. A-D. Confocal micrographs showing two nerve bundles identified by the NFH antibody (B) and two different PGP9.5 antibodies (C, guinea pig polyclonal; D, mouse monoclonal). All the nerve fibers identified by PGP9.5 staining show NFH immunoreactivity. Scale bar, 50 μm.

Confocal microscopic evaluation of pulpal sections from normal wisdom teeth showed the presence of numerous N52-expressing nerve fibers and a comparison of this staining with that obtained with the NFH antibody showed nearly identical staining of the same fibers with both antibodies (Figure 1). These results show that both antibodies (N52 is a mouse monoclonal and NFH is a chicken polyclonal) stain the same population of nerve fibers and therefore either one can be used interchangeably to study staining relationships with other antibodies. A comparison of NFH staining to the staining obtained with two different PGP9.5 antibodies (mouse monoclonal and guinea pig polyclonal), showed that most PGP9.5 fibers also expressed NFH (Figure 2). The same nerve fiber population was stained with both PGP9.5 antibodies. Since PGP9.5 is commonly used to identify all nerve fibers in peripheral tissues [19,20], together these findings show that most nerve fibers within the human dental pulp express NFH/N52, a protein commonly expressed in sensory neurons that give rise to myelinated axons [12].

### Fibers that lack NFH/N52 express TH and are associated with blood vessels

**Figure 3:**
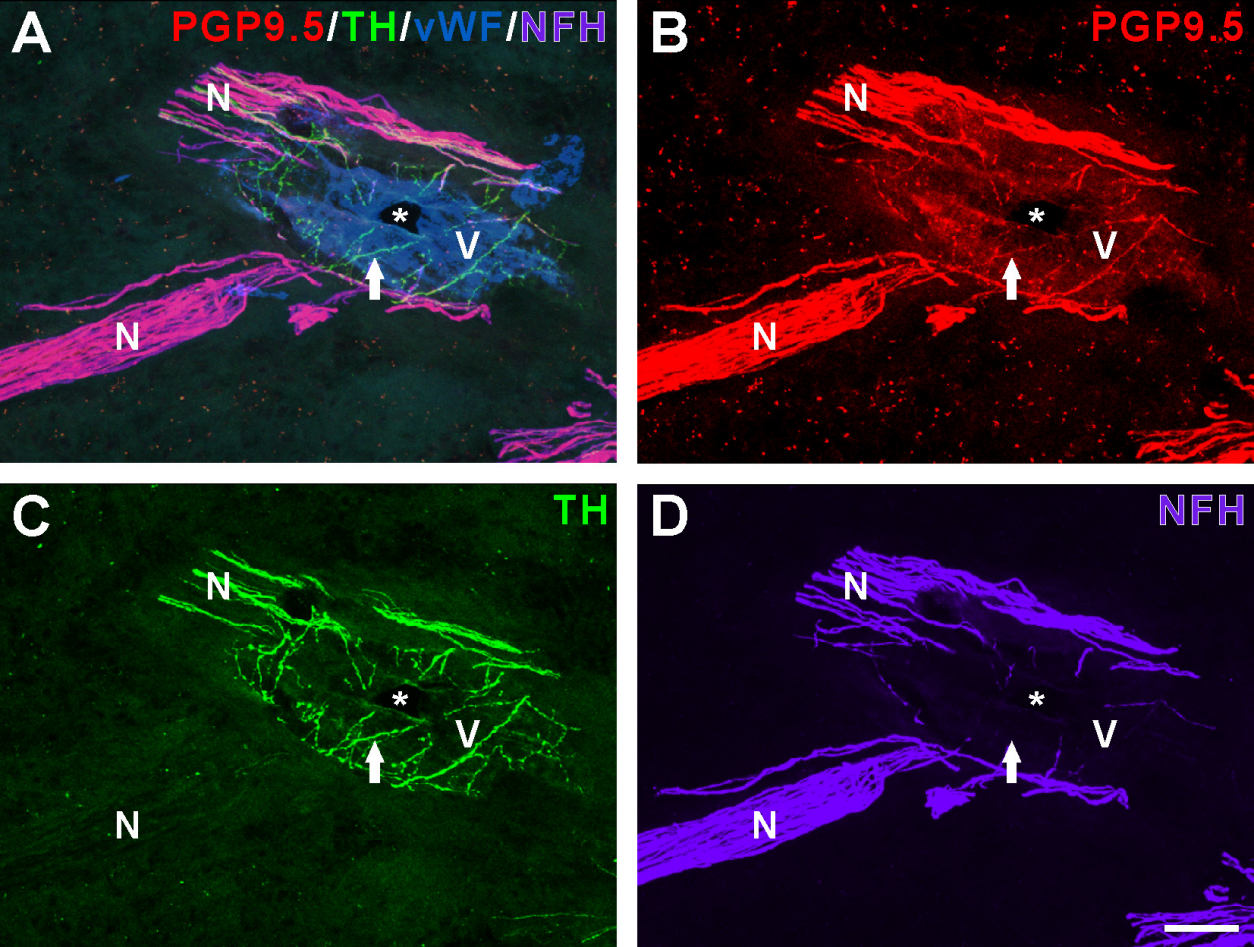
**Sympathetic fibers associated with a blood vessel express PGP9.5 while mostly lacking neurofilament heavy (NFH)**. A-D. Confocal micrographs showing PGP9.5 (B, *red*), tyrosine hydroxylase (C, *green*) and NFH (D, *purple*)-identified nerve fibers associated with a blood vessel identified with von Willebrand Factor (vWF; A, *blue*). The PGP9.5-identified nerve fibers include sympathetic fibers that express TH and fibers with NFH. Fibers with TH are prominently associated with the blood vessel (*V*) and many of these fibers lack NFH (*arrow*). Most of the PGP9.5-identified fibers located within the two nerve bundles (*N*) next to the blood vessel express NFH. The *asterisk *indicates the lumen of the blood vessel. Scale bar, 50 μm.

Although rare, occasionally some PGP9.5-identified nerve fibers lacked NFH/N52 and further characterization identified these fibers as TH positive axons that were mostly seen in close association with vWF-identified blood vessels (Figure 3). These TH-positive fibers most likely represent sympathetic fibers that innervate blood vessels and appear to represent the one major subpopulation of fibers that do not express NFH within the human dental pulp.

### Many fibers with NFH/N52 lack MBP staining

**Figure 4:**
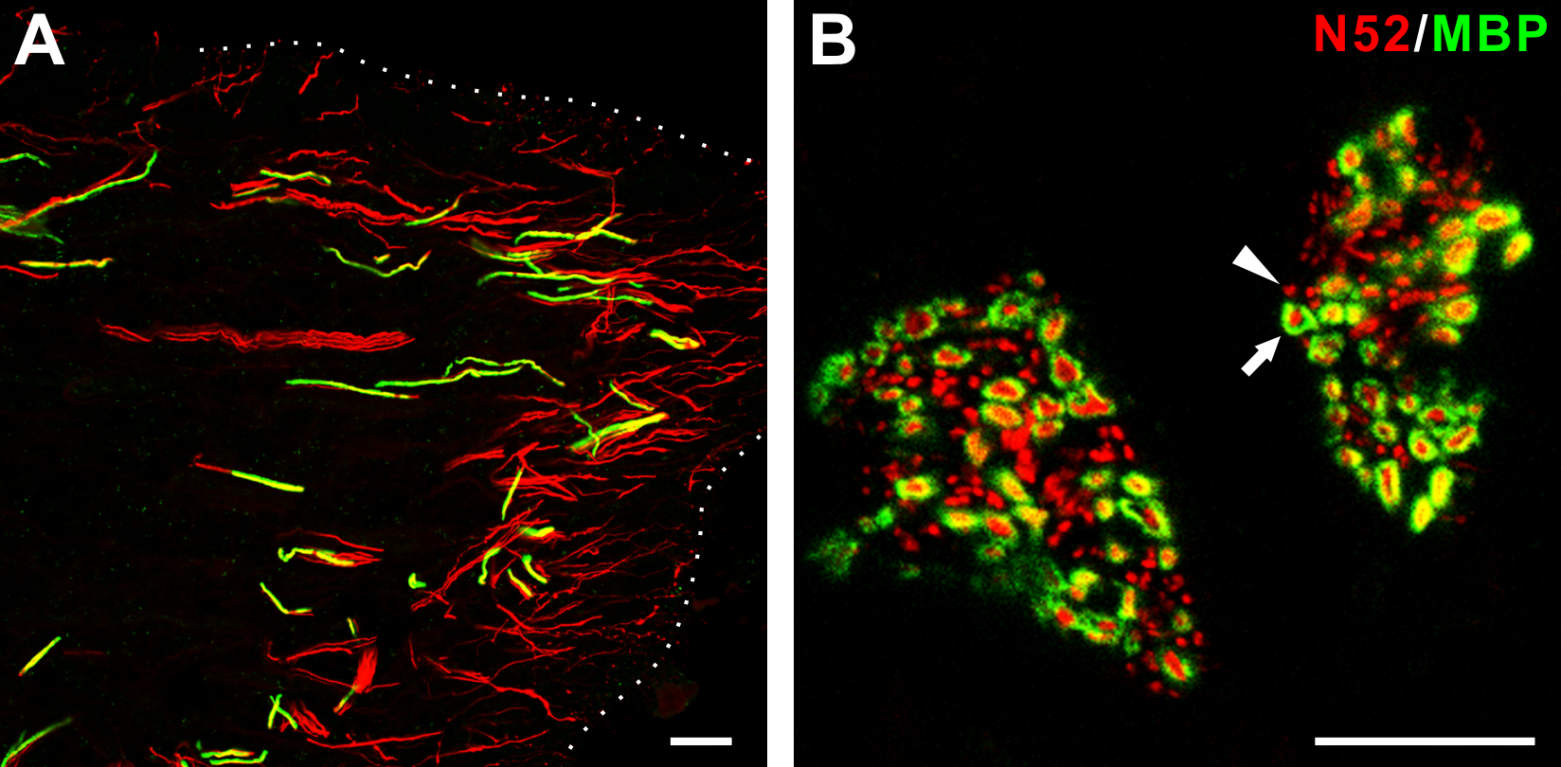
**Many N52-identified nerve fibers lack myelin staining within different regions of the human dental pulp**. A and B. Confocal micrographs showing N52 (*red*) and myelin basic protein (MBP; *green*) staining relationships in the coronal region (A; longitudinal plane) and in nerve fiber bundles located in the center of the radicular area (B; cross-section). Both images show the presence of N52-identified nerve fibers that lack myelin (*arrowhead*) and fibers with myelin (*arrow*). The nerve fibers without myelin are common in both pulpal regions but more so within the coronal region. The outline shown in A indicates the approximate position of the odontoblastic layer. Scale bar, 20 μm.

Additional staining showed that many of the N52-positive axons lacked MBP staining (Figure 4). This lack of MBP was especially prominent within coronal regions and at peripheral locations throughout the pulp where odontoblasts are located (Figure 4A). This staining relationship was also critically examined in other specimens where bundles of axons located in the radicular pulp were seen in cross section and although N52 fibers with MBP staining were more common than seen near the odontoblast layer, many N52-positive axons lacked MBP staining (Figure 4B).

### NaCh clusters are present in NFH/N52 fibers at nodal sites and at sites that lack MBP and caspr

**Figure 5:**
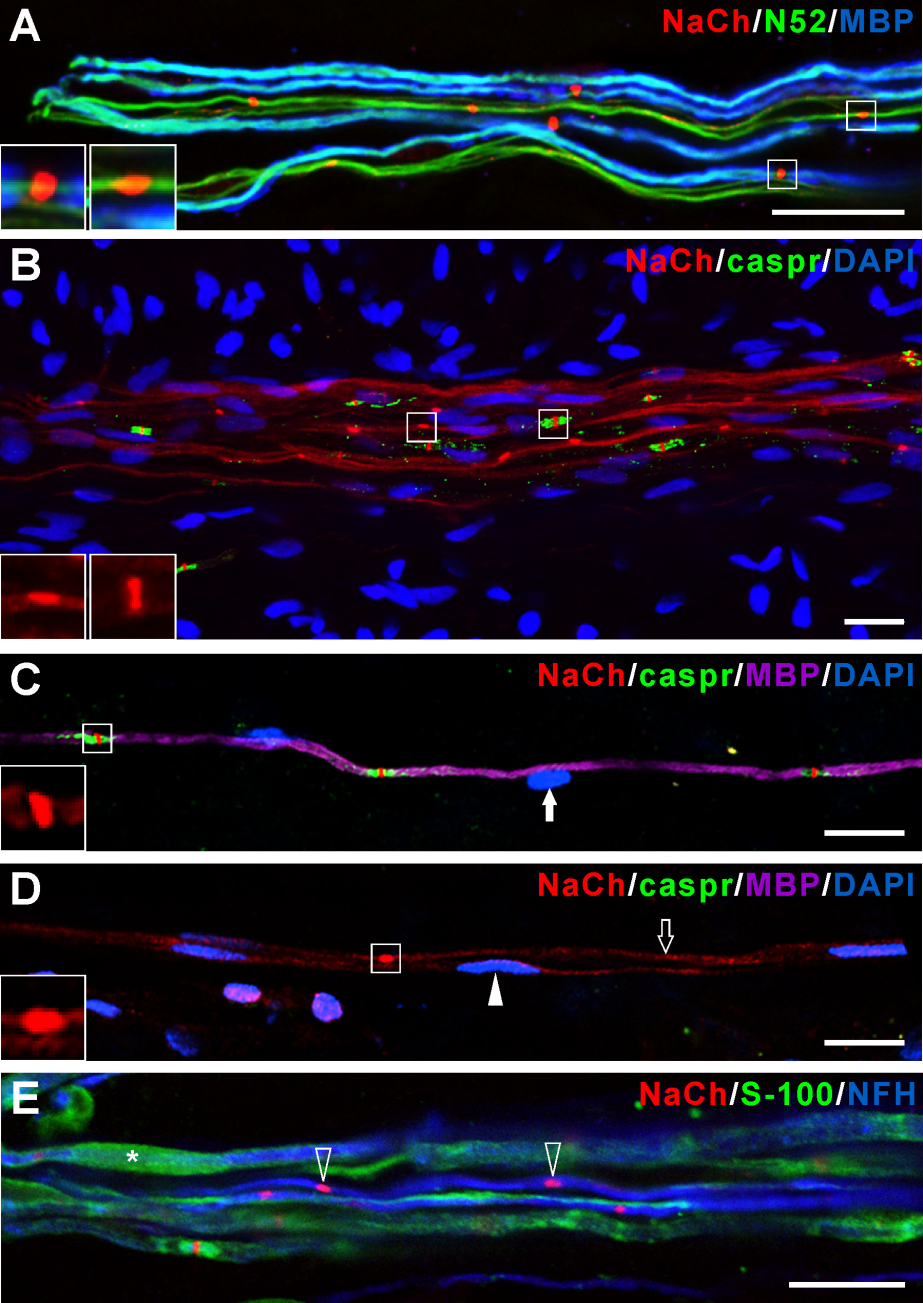
**Sodium channel (NaCh) clusters are present in unmyelinated fibers and some lack associations with Schwann cells**. A. Confocal micrograph showing NaCh (*red*), N52 (*green*), and myelin basic protein (MBP; *blue*) staining relationships. Clusters of NaChs are seen in fibers with and without myelin. The inserts represent enlargements of a typical disc-like nodal NaCh cluster on a myelinated fiber (*left*) and a torpedo-like NaCh cluster in an unmyelinated axon with N52 that lacks MBP (*right*). B. Confocal micrograph showing NaCh (*red*), caspr (*green*), and DAPI (*blue*) staining relationships. Clusters of NaChs are seen at typical caspr-identified nodal sites and at sites that lack caspr. The inserts are enlargements of a typical nodal NaCh cluster flanked by caspr staining (*right*) and a torpedo-like NaCh cluster on an axon that lacks caspr (*left*). C and D. Single confocal micrographs showing NaCh (*red*), caspr (*green*), MBP (*purple*), and DAPI (*blue*) staining relationships. The inserts represent enlargements of a typical nodal NaCh cluster flanked by caspr in a myelinated fiber (C) and a torpedo-like NaCh cluster in an axon that lacks both MBP and caspr (D). The DAPI-identified nucleus associated with the myelinated fiber appears oval (C, *white arrow*), whereas the nucleus associated with the nerve fiber that lacks both MBP and caspr appears flattened (D, *white arrowhead*). The unmyelinated fiber also shows a broad distribution of NaChs in areas beyond the torpedo-like NaCh cluster (D, *black arrow*). E. Single confocal image showing NaCh (*red*), S-100 (*green*), and neurofilament heavy (NFH; *blue*) staining relationships. Clusters of NaChs can be observed on some NFH-positive nerve fibers that lack S-100 staining (E, *white-outlined arrowheads*). S-100 staining can be found in the cytoplasm of Schwann cells wrapping around the axons (E, *Asterisk*). Scale bar, 20 μm.

Specimens stained with the pan-specific NaCh antibody showed the presence of NaCh clusters within N52-positive axons that were associated with MBP, while other N52 positive axons that lacked MBP also showed NaCh clusters (Figure 5A). The overall appearance of the NaCh clusters varied depending on the presence or absence of MBP. In those fibers with MBP staining, the clusters typically appeared disc-like, compact and at times extended radially beyond the region of N52 staining (Figure 5A). In contrast, the NaCh clusters in the N52 fibers without MBP appeared elongated and confined to the region of the axon with N52 staining (Figure 5A). Overall, these clusters within the N52 fibers that lacked MBP appeared torpedo-like (Figure 5A). In an attempt to further characterize the nature of these different NaCh clusters, specimens were stained with caspr (paranodin), a paranodal protein used to identify nodes of Ranvier [21]. Evaluation of these specimens identified the localization of NaCh clusters at caspr-identified typical nodal sites and within fibers at sites that lacked caspr (Figure 5B, C, D). Again, there was a generalized difference in the overall appearance of NaCh clusters as based on the presence or absence of associated caspr staining. Those clusters associated with caspr appeared disc-like (Figure 5B, C) whereas the clusters that lacked caspr appeared more elongated and torpedo-like (Figure 5D), similar to those described earlier in the fibers that lacked MBP staining (Figure 5A). In addition, the localization of NaChs was mostly confined to clusters at nodal sites in the fibers with caspr, whereas nerve fibers that lacked caspr also showed a more even distribution of NaChs along the entire length of the axon that appeared less dense than those present within the clusters located in these same fibers (Figure 5B, D). Another difference included the shape of the nuclei of the cells that were closely associated with single nerve fibers that varied depending on the presence or absence of MBP staining and on the appearance of the NaCh clusters. In general, this difference included nuclei with a more rounded appearance in fibers with MBP and caspr staining (Figure 5C), whereas the nuclei associated with fibers with more elongated-shaped NaCh clusters that lacked caspr and MBP, appeared more flattened (Figure 5D). These nuclei are most likely the nuclei of Schwann cells and these differences may represent variations in the shape of nuclei between myelinating and unmyelinating Schwann cells.

### NaCh clusters are present in fibers that are associated with S-100 and that lack S-100

Other staining was done to further evaluate the relationship of NaCh clusters seen within NFH-identified nerve fibers with S-100 identified Schwann cells. Clusters of NaChs were seen in fibers with NFH that showed an association with S-100 expression and in fibers that lacked this association (Figure 5E). The fibers with associated S-100 staining typically showed S-100 expression within the cytoplasm of processes that enwrapped axons and that was especially prominent in regions adjacent to the paranode of axons. The NaCh clusters seen in fibers associated with S-100 staining appeared disc-like. In contrast, the NaCh clusters located within the NFH-identified nerve fibers that lacked closely associated S-100 staining appeared more elongated and torpedo-like (Figure 5E), similar to those described above.

## Discussion

The results of this study show the common expression of NFH/N52 in most nerve fibers in the human dental pulp and the presence of NaCh clusters within the unmyelinated segments of these same fibers. The broad expression of NFH/N52 within pulpal afferents was also seen in many fibers that lacked a myelin sheath and the combination of this finding with other findings performed in experimental animals (see below) strongly suggests that many of the unmyelinated fibers within the human dental pulp originate from axons that are myelinated at more proximal locations. Moreover, the presence of NaCh clusters within unmyelinated fibers suggests that the unmyelinated segments of myelinated axons show an inherent capacity for NaCh cluster formation. These findings have implications regarding the use of the human dental pulp as a model to study pain mechanisms, the role of Schwann cell-axonal interactions responsible for the NaCh localization within the axolemma, and the possible influence of NaCh clusters on action potential initiation and propagation within unmyelinated axons.

The dental pulp is a common site of disease and this disease is typically associated with pain [1-3]. In fact, the application of various stimuli to either exposed dentin or to pulp tissue generally produces the sensation of pain [6-8]. These findings have led to the use of the dental pulp as a model for the study of pain mechanisms [22]. Numerous studies have used the electron microscope to classify the nerve fiber population present within the dental pulp of various species as based on the presence or absence of myelin and these findings have formed the basis for the different pain sensations experienced with toothache [23]. Results of these studies show unmyelinated fibers typically represent 70-90% of all fibers in the pulp [10,11]. However, the results of these electron microscopic studies contrast sharply with the results of other studies performed in experimental animals that have used retrograde labeling techniques to evaluate the size and the histochemical composition of pulpal sensory neurons within the trigeminal ganglion [24-27]. These results consistently show that pulpal afferents typically have large and medium diameters and these sizes are more consistent with cell bodies that give rise to myelinated afferents rather than unmyelinated ones [12,28,29]. Other findings suggest a thinning of fibers and progressive loss of the myelin sheath as axons course toward the tooth since the proportion of myelinated axons relative to unmyelinated axons is reduced in nerves closer to teeth when compared to more distant sites [30,31]. A progressive loss of myelin is also seen within the tooth since the proportion of unmyelinated axons is greater at more coronal locations than seen near the root apex in rat molars [32]. Additionally, the faster conduction velocities of action potentials recorded in extrapulpal segments when compared to intrapulpal locations is also suggestive of alterations in myelination status [33,34]. More recently, retrograde labeling with horseradish peroxidase showed almost all parent axons innervating the rat molar dental pulp were myelinated within the proximal root of the trigeminal ganglion [33-35]. The common expression of NFH/N52 in pulpal afferents that lack myelin as seen in our study provides additional support for a significant myelinated afferent innervation of the human dental pulp. Together, these findings suggest that the classification of the nerve fiber population present within peripheral tissues by anatomical methods that evaluate axon diameter or the presence or absence of myelin may not be representative of the actual population of neurons that give rise to these same fibers. Furthermore, the myelinated origin of the unmyelinated fibers should be considered when using the dental pulp to examine pain mechanisms.

The pain sensations that follow the stimulation of the pulpodentin complex or those experienced with toothache include both sharp-shooting and dull-ache sensations and the results of human studies show these are due to a select activation of A-delta and c-fibers, respectively [13-15]. Experimental animal studies have also recorded action potentials with conduction velocities that are consistent with the presence of both unmyelinated and myelinated axons that innervate the dental pulp [23], but some of the fibers with c-fiber conduction velocities may actually represent the unmyelinated segments of fibers with myelin at more proximal locations. This possibility suggests the activation of some unmyelinated fibers present within the dental pulp may give rise to sensations that are more typical of myelinated fibers.

The identification of NaCh clusters within normal fibers that lack myelin in tissues without pathology represents a novel finding. Similar NaCh clusters, including those with a torpedo-like morphology, have only been described previously within acutely demyelinating axon segments or in large diameter axons that lack myelin in dystrophic mice [36,37]. The presence of such clusters may contribute to axonal excitability and theoretical modeling calculations suggest the activation of NaCh clusters in unmyelinated axons could increase the efficiency of action potential propagation [38].

An intriguing aspect related to the presence of NaCh clusters within unmyelinated fiber segments concerns the molecular signaling mechanisms responsible for this clustering. Nodes of Ranvier and the initial segments of axons both contain a high density of NaChs, yet the signaling mechanisms responsible for this targeting within these specific locations appear to differ. The clustering at nodes depends on signals provided by myelinating glia, whereas the localization within axon initial segments is independent of these extrinsic signals and instead appears as an inherent property of the neuron [39-41]. In this regard, the torpedo-shaped NaCh clusters seen within the unmyelinated segments of NFH/N52 axons appear to be independent of cues provided by the direct contact of myelinating glia since they were seen in axons that lacked staining for myelin basic protein. Evidence to support an intrinsic ability of axons to form NaCh clusters includes clusters seen in zebrafish mutants lacking Schwann cells [42] and in axons of rat retinal ganglion cells when grown in culture and lacking direct glial contact [43,44]. However, it is possible that some cues may be provided by adjacent unmyelinating Schwann cells, or that diffusible factors released from myelinating Schwann cells on adjacent axons could have induced such ectopic clusters to form (Figure 5E). Furthermore, it is possible that the unmyelinated axons of only a certain class of sensory neurons show the inherent ability to cluster NaChs and this ability may be greater in neurons that give rise to myelinated axons, such as the ones broadly expressed within the human dental pulp. The finding of NaCh clusters within the unmyelinated segments of pulpal afferents suggests some axons have the intrinsic ability to cluster NaChs independent of cues from myelinating glia and the dental pulp represents one possible site to further evaluate these issues.

We have previously examined the pan-specific, Nav1.6 and 1.7 NaCh isoform expressions in human dental pulp specimens isolated from extracted normal wisdom teeth and compared these expressions to those seen in molar teeth with pulpitis [16-18]. These studies used quantitative image analysis techniques to evaluate NaCh expressions within single nerve fibers at caspr-identified nodal sites. Results from these studies consistently showed a prominent demyelinating response of axons within the pulpitis samples that resulted in the increased incidence of atypical nodal forms. Our study with the pan-specific NaCh antibody included a quantification of NaCh clusters that lacked caspr and our results showed these "naked" NaCh accumulations were present in both normal and painful specimens [16]. Even though some of these "naked" clusters most likely result from the loss of myelin, their common occurrence in normal specimens as seen in our pan-specific NaCh study and the findings presented here provide additional evidence for their existence in unmyelinated fibers. Our studies that evaluated changes in Nav1.6 and 1.7 expressions did not specifically examine these naked clusters, so the isoforms present in these naked clusters is unknown. Further characterization of the specific isoforms expressed within these naked clusters and possible changes in expression in pain conditions are needed since some isoforms may be preferentially involved. A preferential involvement of specific isoforms within NaCh clusters in pain conditions is important since this could represent one mechanism contributing to increased activation of nociceptors.

## Conclusions

The human dental pulp is richly innervated by unmyelinated nerve fibers and historically much of the pain associated with toothache was thought to result from activation of small diameter neurons that typically give rise to c-fibers. Our results and the results of others suggest that many of these unmyelinated nerve fibers actually originate from myelinated fibers and therefore much of the pain associated with toothache may actually involve the activation of larger diameter neurons. The thinning of fibers due to myelin loss appears as a prominent feature of pulpal afferents that may represent a unique phenotype. We also identified NFH/N52 in many fibers that lacked myelin and therefore the presence of NFH/N52 alone, especially in peripheral tissues, does not necessarily equate to the presence of myelin. Lastly, the identification of NaCh accumulations within unmyelinated fibers was unexpected and has broad implications related not only to axonal excitability, but also the intrinsic ability of axons to cluster NaChs in the absence of molecular clues from myelinating glial cells. The dental pulp appears as an especially attractive model to further evaluate these issues.

## Methods

### Human dental pulp collection and preparation

This study was approved by the Human Subjects Institutional Review Board at the University of Texas Health Science Center at San Antonio. Informed consent was obtained from all human subjects who participated in this study. Teeth included in this study were limited to normal third molar (wisdom) teeth with fully formed apices that were previously scheduled for extraction. A total of twenty teeth were collected from twenty patients (one tooth/patient). All teeth lacked the presence of a carious lesion or a past history of pain. Extracted teeth were placed in 0.1 M phosphate buffer (PB). Later the same day, the teeth were split longitudinally and the pulpal tissues were removed and fixed in 4% paraformaldehyde in 0.1 M PB for 30 minutes. The pulpal tissue was rinsed in 0.1 M PB and then placed in 30% sucrose in 0.1 M PB overnight at 4°C. The next day the pulp was embedded in Neg-50 (Richard-Allan Scientific; Kalamazoo, MI) and serially sectioned with a cryostat at 30 μms in the longitudinal plane or cut in cross section. Sections were placed onto Superfrost Plus slides (Fisher Scientific, Pittsburgh, PA), air dried and then stored at -20°C.

### Antibodies

All primary antibodies used in the present study are summarized in Table 1 and described in more detail below.

**Table 1:** List of Antibodies

Name	Manufacturer	Catalog#/Clone	Host	Type	Dilution	Purpose
Neurofilament 200 kD (N52)	Sigma-Aldrich, St. Louis, MO	N0142 Clone N52	Mouse	Mono-clonal	1:2000	Identify subset of nerve fibers that express NFH

Neurofilament Heavy (NFH)	Abcam, Cambridge, MA	AB4680	Chicken	Poly- clonal	1:1000	Identify subset of nerve fibers that express NFH

Protein gene product 9.5 (PGP9.5)	Millipore, Billerica, MA	AB5898	Guinea pig	Poly- clonal	1:500	Identify all nerve fibers

Protein gene product 9.5 (PGP9.5)	UltraClone Limited, UK	Clone 31A3	Mouse	Mono- clonal	1:100	Identify all nerve fibers

Tyrosine hydroxylase (TH)	Millipore, Billerica, MA	MAB318 Clone LNC1	Mouse	Mono- clonal	1:100	Identify sympathetic nerve fibers

Von-Willebrand factor (vWF)	Dako, Denmark	A0082	Rabbit	Poly- clonal	1:2000	Identify endothelial cells

Myelin basic protein (MBP)	Millipore, Billerica, MA	MAB386 Clone 12	Rat	Mono- clonal	1:500	Identify myelin associated with myelinated fibers

Sodium channel (pan NaCh)	Rock Levinson's lab	EOIII	Rabbit	Poly- clonal	1:100	Identify all NaCh isoforms

Contactin-associated protein 1 (caspr)	Elior Peles's lab	Clone 275	Mouse	Mono- clonal	1:500	Labels paranode region; used to identify nodes of Ranvier

S100	ABR-Affinity BioReagents, Golden, CO	MA1-23594 (Clone B32.1)	Mouse	Mono- clonal	1:500	Identify Schwann cells

List of antibodies including name, manufacturer, catalog number &/or clone number, host, type, dilution and purpose that were used in this study.

Mouse monoclonal anti-neurofilament (N52) 200 kDa antibody has been extensively characterized in previous studies [45,46]. This antibody shows a wide range of species reactivity including humans [17,47]. The staining pattern seen in the current study is consistent with that seen previously [17,47,48].

Chicken polyclonal anti-neurofilament heavy (NFH) 200 kDa antibody shows cross reactivity in a wide range of species including humans. This antibody identifies specific immunoreactivity in neurons in the rat peripheral nervous system and in skin nerve endings as shown previously [49,50]. The staining pattern seen with this antibody in the current study is similar to that seen with the mouse monoclonal N52 antibody and consistent with that seen before [49].

Guinea pig polyclonal anti-protein gene product (PGP) 9.5 antibody has been used in many previous studies [51-53] and specifically identifies neuronal cell bodies and nerve fibers in a wide range of species including humans. The staining pattern seen in the current study is consistent with that seen previously with this particular antibody and with other PGP9.5 antibodies [53-55].

Mouse monoclonal anti-PGP9.5 antibody and a rabbit polyclonal anti-PGP9.5 antibody (both from Ultraclone and raised against the same antigen) have been used in many previous studies [55-57] and consistently labels neuronal cell bodies and nerve fibers. The staining pattern seen in the current study is consistent with previous studies that used the same PGP9.5 antibodies [55-57] and also consistent with the staining pattern seen with the guinea pig polyclonal PGP9.5 antibody used in this study.

Mouse monoclonal anti-tyrosine hydroxylase (TH) antibody shows reactivity in a wide range of species including humans and specifically labels sympathetic nerve fibers in the rat DRG. This antibody has been used in many previous studies [58-61] and the staining pattern seen in the current study is consistent with other studies that used the same or other TH antibodies [62,63].

Rabbit polyclonal anti-von Willebrand Factor (vWF) antibody has been used extensively in previous studies to identify blood vessels and to characterize endothelial cells [64-66]. The staining pattern seen in the current study is consistent with other studies [65,66].

Rat monoclonal anti-myelin basic protein (MBP) antibody shows a wide range of species reactivity including humans. This antibody has been used extensively in previous studies to identify compact myelin sheath [67-69] and the staining pattern seen in the current study is consistent with other studies that used the same or different MBP antibodies [67,70].

Rabbit polyclonal anti-sodium channel (NaCh) antibody identifies a conserved epitope located within the alpha subunit of all NaCh isoforms and so is used as a pan-specific antibody to identify all NaCh isoforms [71]. This antibody has been widely used in previous studies to identify NaCh clusters at nodes of Ranvier in both CNS and PNS and the staining pattern seen in the current study is consistent with other studies [16,39,40,72].

Mouse monoclonal anti-contactin-associated protein (caspr; also known as paranodin) was kindly provided by Dr. Elior Peles. This antibody has been used extensively in previous studies [16,73-75] and consistently shows specific staining of the paranodal region and is used to identify nodes of Ranvier. The staining pattern in the current study is consistent with other studies [16,73-75].

Mouse monoclonal anti-S100 antibody was used to identify Schwann cells. This clone (B32.1) has been used extensively in previous studies [76-78] and the staining pattern of Schwann cells seen in the current study is consistent with that identified by other S100 antibodies in other studies [71,79].

### Immunohistochemistry

Immunostaining was performed as described previously [72]. Tissue sections were permeabilized and non-specific protein binding sites were blocked with blocking solution consisting of 4% normal goat serum (Sigma, St. Louis, MO, USA), 2% bovine gamma globulin (Sigma), and 0.3% Triton X-100 (Fisher Scientific) in 0.05 M phosphate buffer saline (PBS) for 90 minutes prior to incubation with primary antibodies diluted in blocking solution for 16 hours. Slides were rinsed with PBS, incubated with species-specific Alexa-Fluor secondary antibodies raised in goat (Molecular Probes, Eugene, OR, USA) for 90 minutes at a 1:100 dilution, rinsed, air dried and coverslipped with Vectashield or Vectashield with DAPI (as a nuclear stain; both from Vector Laboratories, Burlingame, CA, USA). All staining procedures described above were performed at room temperature.

### Microscopy, image acquisition and immunohistochemistry controls

Digital images were acquired with a Nikon D90-Eclipse microscope and a C1si laser scanning confocal imaging system equipped with 4 solid state lasers (408 nm, 488 nm, 561 nm and 638 nm) with either a 20×/0.75 N or a 40×/1.30 N objective lens (Nikon Corp.). Images were processed for illustration purposes with Adobe Photoshop CS2 (Adobe Systems, San Jose, CA) and CorelDRAW 12 (Corel Corporation, Ottawa, Canada). Control preparations consisted of tissue sections that were processed as above but that lacked either primary and secondary antibodies or primary antibodies and that were examined with identical laser gain and other settings as those used to capture optical images in the experimental sections. Optical images obtained from these control preparations showed a lack of immunofluorescence in the specific structures identified with the primary antibodies described above.

## Authors' contributions

MAH conceived the study, participated in its design, assisted in microscopic evaluation of stained tissue specimens, assisted in acquisition and interpretation of images, and drafted the manuscript. SL assisted with tissue preparation, performed tissue staining, microscopic evaluation of stained tissue specimens, image acquisition, assisted in interpretation of images, and helped draft the manuscript. SRL helped to conceive the study, produced the NaCh antibody, assisted with interpretation of images, and helped draft the manuscript. All authors read and approved the final manuscript.
